# An Electrokinetically-Driven Microchip for Rapid Entrapment and Detection of Nanovesicles

**DOI:** 10.3390/mi12010011

**Published:** 2020-12-24

**Authors:** Leilei Shi, Leyla Esfandiari

**Affiliations:** 1 Department of Electrical Engineering and Computer Science, College of Engineering and Applied Sciences, University of Cincinnati, Cincinnati, OH 45221, USA; shili@mail.uc.edu; 2 Department of Biomedical Engineering, College of Engineering and Applied Sciences, University of Cincinnati, Cincinnati, OH 45221, USA

**Keywords:** nanovesicle, biological nanoparticles, exosome, impedance, dielectrophoresis, biosensing, microfluidics, lab-on-a-chip

## Abstract

Electrical Impedance Spectroscopy (EIS) has been widely used as a label-free and rapid characterization method for the analysis of cells in clinical research. However, the related work on exosomes (40–150 nm) and the particles of similar size has not yet been reported. In this study, we developed a new Lab-on-a-Chip (LOC) device to rapidly entrap a cluster of sub-micron particles, including polystyrene beads, liposomes, and small extracellular vesicles (exosomes), utilizing an insulator-based dielectrophoresis (iDEP) scheme followed by measuring their impedance utilizing an integrated electrical impedance sensor. This technique provides a label-free, fast, and non-invasive tool for the detection of bionanoparticles based on their unique dielectric properties. In the future, this device could potentially be applied to the characterization of pathogenic exosomes and viruses of similar size, and thus, be evolved as a powerful tool for early disease diagnosis and prognosis.

## 1. Introduction

Electrical Impedance Spectroscopy (EIS) has been desirable for the characterization of various biological entities, including various cell types, waterborne parasites, bacteria, and bacteria spores, since it can be used as a label-free method with minimal sample preparation procedure [1,2,3,4,5,6,7,8,9,10,11,12]. This technique has been used to differentiate various cell types and to identify abnormal or tumor cells [13,14]. One popular design for EIS is the single cell impedance cytometry, in which a pair of facing or coplanar electrodes are embedded in a microfluidic channel [2,3,5,15,16,17,18,19]. The electrodes are energized with a voltage at one or more discrete frequencies, generating an electric field within the channel. As a single cell passes through the microfluidic channel, the fluctuation of the electric current is detected, and thus, provides the impedance of a single cell. Another strategy is based on the static state impedance measurement approach, in which a single cell is manipulated to be placed at the center of the electrodes, and thus, the electric field in the detection volume is altered due to the presence of the cell [4,14,20]. However, the related work on small extracellular vesicles (exosomes) with diameters of 40–150 nm and the particles of similar size has not yet been reported. The main challenge for adapting this system for analysis of a single vesicle is that the scale of the channel and/or electrodes must be miniaturized to the corresponding size scale of the target vesicle in order to achieve a reliable sensitivity [4]. Although the device with a miniaturized channel and electrodes could be fabricated, it is very challenging to pass a single vesicle, one at a time, through the channel or manipulate it to the designated position. In addition, a high applied pressure would be needed to overcome the high resistance of the submicron channel to omit the channel’s blockage by the vesicles. 

Dielectrophoresis (DEP) has been utilized for isolating and manipulating micro-/nano-scale particles and biomolecules due to its rapid and label-free criteria [21,22,23,24,25]. Moreover, we have previously demonstrated that a new insulator-based dielectrophoretic (iDEP) device made of an array of micropipettes can be utilized for rapid entrapment of nanovesicles based on their unique dielectric properties at pipettes’ pores, which is due to the balance of three electrokinteic forces including dielectrophoretic (DEP), electrophoretic (EP), and electroosmotic (EOF) forces [26,27]. In this paper, we have fabricated a microchip to rapidly entrap a cluster of vesicles utilizing an iDEP scheme by applying a direct current (DC) followed by simultaneously measuring their impedance by embedded microelectrodes and applying an alternative current (AC) at a wide frequency spectrum. By using this method, the sub-micron particles could be entrapped in a micro-scale trapping zone and be detected by impedance probing without losing the sensitivity and facing the blockage issue. In addition, electrolyte solutions with different ionic strengths with and without suspended particles have been utilized to study the capability of the device to differentiate between nanoparticles with different dielectric properties. The microchip was able to differentiate between various sub-micron particles of similar size, including polystyrene beads, liposomes, and exosomes and, thus, it has the potential to be further evolved as a characterization tool for differentiation of circulating nanovesicles for diagnostic purposes.

## 2. Materials and Methods 

### 2.1. Materials

All chemicals were purchased from Sigma-Aldrich (St. Louis, MO, USA) unless otherwise noted. Carboxylic acid polystyrene (COOH-PS) beads (100 nm) were obtained from Phosphorex Inc. (Hopkinton, MA, USA). N-(7-nitrobenz-2-oxa-1,3-diazol-4-yl)-1,2-dihexadecanoyl-sn-glycero-3-phosphoethanolamine (NBD-DHPE) fluorescently labeled 100 nm liposomes were purchased from FormuMax Scientific Inc. (Sunnyvale, CA, USA). Telomerase reverse transcriptase (hTERT) Mesenchymal Stem Cell Exosomes with the average diameter of 146 nm were purchased from ATCC (Manassas, VA, USA). The zeta potential and the size distribution for all the particles have been shown in Table S1. Silicone elastomer base and curing agent were purchased from Dow Corning (Elizabethtown, KY, USA). Gold etchant (Type TFA) and chromium etchant (1020AC) were obtained from Transene Company Inc. (Danvers, MA, USA). Photoresist AZ5214E and developer AZ917 MIF were purchased from Integrated Micro Materials (Argyle, TX, USA). SU-8 2050, SU-8 developer, and OmniCoat were obtained from Microchem Corp. (Westborough, MA, USA). Polyimide PI2610 and adhesion promoter MV652 were obtained from Hitachi DuPont MicroSystems LLC. (Parlin, NJ, USA). Heat seal connectors were obtained from Elform Inc. (Reno, NV, USA). The home-designed printed circuit board (PCB) was fabricated by PCB Universe (Vancouver, WA, USA). Glass slides were purchased from Ted Pella Inc. (Redding, CA, USA).

### 2.2. Preparation of Sub-Micron Particles

Electrolyte solutions containing different potassium chloride (KCl) concentrations (10, 100, and 500 mM) were prepared at pH 7.0. The conductivity of KCl solutions were measured utilizing a conductivity meter (Oakton Cond 6+) as: 0.3 S/m for 10 mM KCl, 1.4 S/m for 100 mM KCl, and 5.9 S/m for 500 mM KCl.

COOH-PS beads (100 nm) were re-suspended into 10 mM KCl to the final concentration of 1.8 × 10^8^/mL and 2.3 × 10^12^/mL. The 100 nm liposomes were re-suspended into 10 mM KCl at a final concentration of 1.9 × 10^11^/mL. Then, 146 nm hTERT Mesenchymal Stem Cell Exosomes were distributed in 10 mM KCl with the concentration of 6.1 × 10^9^/mL. The zeta potential of COOH-PS beads, liposomes, and exosomes dispersed in 10 mM KCl at 25 °C were measured at least 3 times using the Zetasizer-NanoBrook Omni (Brookhaven Instruments, Holtzville, NY, USA).

### 2.3. Device Layout and Fabrication

**Figure 1:**
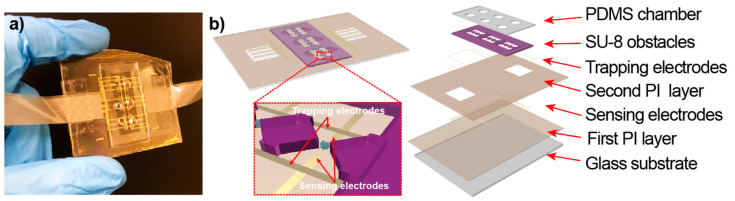
(**a**) The picture of the Lab-on-a-Chip (LOC) device. (**b**) Schematic of the LOC device including the insulator-based dielectrophoresis (iDEP) module for particle trapping (polydimethylsiloxane (PDMS) chamber, SU-8 obstacles, and Trapping electrodes) and the impedance sensing module (Sensing electrodes).

The Lab-on-a-Chip (LOC) device was designed with AutoCAD 2018. The picture of the LOC device was shown in Figure 1a, and a cross-sectional view of the LOC device was shown in Figure 1b. The device contained seven layers as follow: the glass substrate, the first polyimide (PI) layer to improve the adhesion strength of the substrate, the sensing electrodes, second PI layer to avoid short circuit of different electrode layers, the trapping electrodes, the SU-8 obstacles, and the polydimethylsiloxane (PDMS) chambers.

**Figure 2:**
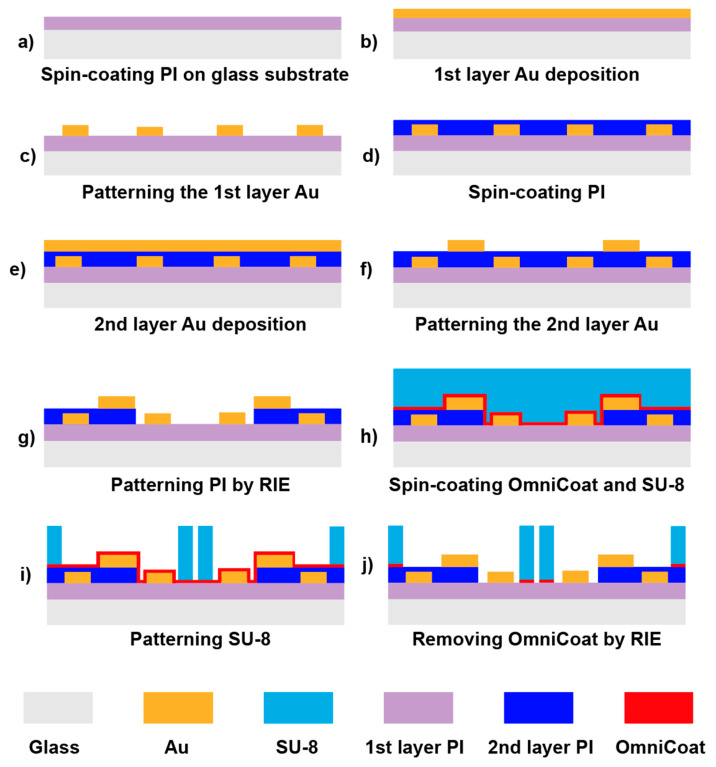
Step-by-step fabrication procedure of proposed LOC device.

The first PI layer was deposited to increase the adhesion between gold and the glass substrate. Prior to the deposition of PI, adhesion promoter VM652 was spin-coated at 2000 rpm for 30 s. PI2610 was then spread at 500 rpm for 5 s followed by 5000 rpm for 30 s to form a 1 μm thin film (Figure 2a). To fabricate the sensing electrodes, a layer of metal (10 nm Cr and 200 nm Au) was deposited on the PI-coated substrate using the E-beam evaporator (Figure 2b). The deposited metal was patterned using the photolithography technique with AZ5214E as the positive photoresist and MIF 917 as the developer. A pair of digital sensing electrode arrays was then created by etching the redundant Au and Cr on the first metal layer. Afterwards, the photoresist residual was removed by acetone (Figure 2c). Prior to the deposition of the trapping electrodes, adhesion promoter VM652 and PI2610 were spin-coated to insulate the sensing electrodes (Figure 2d). Then, 10 nm Cr and 200 nm Au were then deposited (Figure 2e) and patterned (Figure 2f). EVG620 mask aligner was used to align the trapping and the sensing electrodes. The width and the length of each trapping electrode was designed to be 0.25 and 26 mm, and the distance between the trapping electrodes was 2 mm. In order to connect the sensing electrodes with the digital impedance analyzer (HF2LI, Zurich Instrument, Zurich, Switzerland), the PI film that covered the corresponding area was removed by a reactive ion etching (RIE) process with the photoresist AZ5214 as the shadow mask. After the pattern was properly defined, two large rectangular windows (9 mm × 8.5 mm) on the sides and a narrow rectangular window (34 μm × 23 mm) in the middle of the device were etched utilizing RIE process (Technics 85 Reactive Ion Etcher, 190 mTorr, 150 W, 6 min) to expose the tails and tips of the sensing electrodes, respectively (Figure 2g). 

Moreover, to develop the obstacles, as trapping zones, a layer of negative photoresist SU-8 2050 was spin-coated at 3000 rpm for 30 s to obtain a 50 μm film (Figure 2h). Prior to SU-8 coating, a thin layer of OmniCoat was spin-coated at 3000 rpm for 30 s to allow easy stripping of SU-8 and improve the adhesion. The SU-8 layer was exposed under 160 mJ/cm^2^ ultraviolet light with a mask and developed with SU-8 developer to create triangular obstacles with 10 μm width separation (Figure 2i). RIE was then performed to remove the residual OmniCoat (Figure 2j). A polydimethylsiloxane (PDMS) chamber was created by pouring the mixture of silicone elastomer base and curing agent (volume ratio 10:1) on a glass slide and heating to 70 °C for 4 h. After the PDMS was fully crosslinked, it was peeled off from the glass slide and cut into rectangular pieces that were 2 cm in width and 4 cm in length. Six holes, with diameters of 3.5 mm, were punched as the inlets and outlets. At the final stage, the PDMS chamber was adhered on the device to cap the SU-8 obstacles and create the opening with the dimension of 10 μm × 50 μm. A heat seal connector was used to connect the tail of the electrodes on the microchip to a home-designed PCB board. The PCB board was then connected to the power supply and the digital impedance analyzer to apply voltage and conduct the impedance measurement.

### 2.4. Particle Trapping and Impedance Measurement

A volume of 25 μL of electrolyte solution containing different particles including 1.8 × 10^8^/mL and 2.3 × 10^12^/mL COOH-PS beads, 1.9 × 10^11^/mL liposomes, and 6.1 × 10^9^/mL exosomes were injected in to different device chambers. A 5 V/mm DC bias was applied across the trapping electrodes using a Keithley 2220 G-30-1 voltage generator for 5 min. The microscopic images were recorded using an inverted microscope, Olympus IX71, equipped with a high-resolution camera, Andor NeoZyla 5.5.

Impedance measurement was conducted utilizing the digital impedance analyzer (HF2LI, Zurich Instrument, Zurich, Switzerland) as an AC field with a peak amplitude of 100 mV swept from 1 kHz to 10 MHz to record the magnitude and phase components at each frequency. Afterwards, the data was processed with a custom script written in MATLAB (MathWorks Inc., Natick, MA, USA) for statistical analysis. The impedance signals were recorded at a sampling rate of 225 samples/s. Each measurement was repeated at least 3 times. Furthermore, to rule out the effect of the particles concentration and to demonstrate the difference between the particle’s dielectric properties, the impedance was normalized based on the ‘opacity’ concept which was reported by Gawad et al. (Equation (1)) [15,28,29]:

(1) $$\begin{array}{c} O_{f}=\frac{Z\left(f\right)}{Z\left(0.5\;MHz\right)} \end{array}$$

where Z(f) and Z(0.5 MHz) are the impedance magnitude measured at frequencies higher than 0.5 MHz and at 0.5 MHz, respectively. This has been widely applied in cell cytometry to normalize the impedance with respect to the cell size and position since the impedance at 0.5 MHz typically reflects the particle size information [5,13,29,30].

The impedance sensitivity has been analyzed based on the following equation [31]:

(2) $$\begin{array}{c} S=\frac{\left|\Delta \tilde{Z}\right|}{\left|{\tilde{Z}}_{m}\right|}=\frac{\left||{\tilde{Z}}_{mix}\left|-\right|{\tilde{Z}}_{m}|\right|}{\left|{\tilde{Z}}_{m}\right|} \end{array}.$$

where $\Delta \tilde{Z}$ is the impedance change due to the presence of particles, ${\tilde{Z}}_{m}$ is the complex impedance of the detection volume containing medium, and ${\tilde{Z}}_{mix}$ is the complex impedance of the mixture (the medium and the particles ) in the detection volume.

Statistical analysis was performed using the student’s *t*-test and two-way analysis of variance. Difference with *p*-values less than 0.05 were considered significant.

After impedance measurements, the device was cleaned by the established Lab-on-Chip device cleaning protocol [32]. Specifically, the device was injected with DI water to push most of the particles out of the channel. Afterwards, the device was soaked in the mild detergent solution, methanol, acetone, and DI water for five minutes each with an ultrasonic bath environment to completely remove the residue. 

### 2.5. Finite Element Analysis 

Finite-element software, COMSOL Multiphysics 5.2a (COMSOL Inc., Burlington, MA, USA), was utilized to determine the distribution of the electric field gradient as 5 V/mm DC was applied across the gap which was created by SU-8 obstacles. The height of the SU-8 obstacles was 50 μm and the gap distance between a pair of triangular SU-8 obstacles was 10 μm. The conductivity and relative permittivity of the suspending solution in the model was set as 0.3 S/m and 80 to mimic the conductivity of 10 mM KCl solution. The temperature and pressure were assumed to be 298 K and zero Pa, respectively. 

The migration mobility of ionic species (u) was computed using the Nernst–Einstein relation (3):

(3) $$\begin{array}{c} u=\frac{D_{i}}{RT} \end{array}$$

in which, $D_{i}$ is the diffusion coefficient, R is the molar gas constant and T is the absolute temperature. For 10 mM KCl, the value of $D_{i}$ was set as 2 × 10^−9^ m^2^·s^−1^.

Boundary conditions corresponding to the solution obtained from the Poisson–Boltzmann equation for electric potential were applied. The boundary conditions established that the electric potential was not diverged and the gradient of this potential on the SU-8 surface varied with the change in surface charge density [33].

### 2.6. Theoretical Modeling and Equivalent Circuit

**Figure 3:**
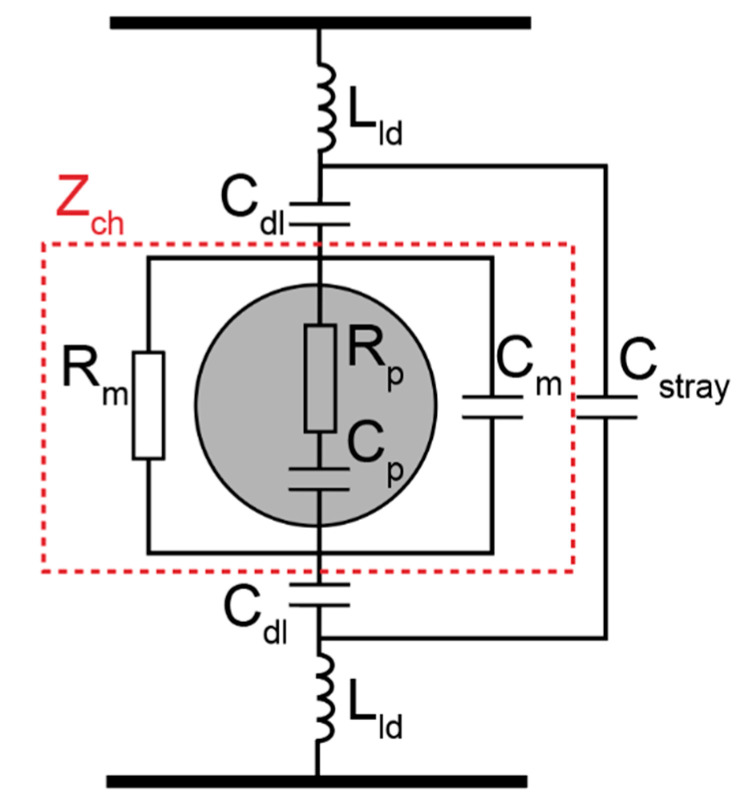
An equivalent circuit model for the impedance measurement system.

A simplified equivalent circuit model (Figure 3) was used to demonstrate the physical principle of the impedance measurement system [34,35,36]. In this model, the channel impedance *Z_ch_* is in series with an electrical double layer capacitance *C_dl_* and is in parallel with a stray capacitance *C_stray_* [34,35,36]. In addition, a lead inductance (*L_ld_*) was included in the equivalent circuit, which is associated with the electrodes and the cables connecting the device to the impedance analyzer [37,38]. The values of *C_dl_*, *C_stray_*, and *L_ld_* were obtained via measurements on electrolyte solutions with well-known electrical properties, followed by fitting into the combination of constant phase element and Cole–Cole model [38,39]. Fitting parameters that were used throughout this theoretical modeling were *C_dl_* = 10 pF, *C_stray_* = 2.2 pF, and *L_ld_* = 6 μH, respectively. 

Channel impedance *Z_ch_* was calculated based on Maxwell’s mixture theory (Equation (4)) [17,40]:

(4) $$\begin{array}{c} {\tilde{Z}}_{ch}=\frac{1}{j\omega {\tilde{\varepsilon }}_{mix}G_{f}} \end{array}$$

where ${\tilde{\varepsilon }}_{mix}$ is the equivalent complex permittivity of the mixture of particles and the medium, ω is the angular frequency, and $G_{f}$ is the geometrical constant of the system.

The equivalent complex permittivity of mixture of homogeneous spherical particles in suspension can be calculated as:

(5) $$\begin{array}{c} {\tilde{\varepsilon }}_{mix}={\tilde{\varepsilon }}_{m}\frac{1+2\phi {\tilde{f}}_{CM}}{1-\phi {\tilde{f}}_{CM}} \end{array}$$

where ϕ is the volume fraction (the volume ratio between the particle and the suspending system), which is estimated as 0.1 for COOH-PS based on the estimated size of entrapped particles cluster under the microscopy; ${\tilde{f}}_{CM}$ is the complex Clausius–Mossotti factor, which is defined as:

(6) $$\begin{array}{c} {\tilde{f}}_{CM}=\frac{{\tilde{\varepsilon }}_{p}-{\tilde{\varepsilon }}_{m}}{{\tilde{\varepsilon }}_{p}+2{\tilde{\varepsilon }}_{m}} \end{array}$$

where ${\tilde{\varepsilon }}_{m}$ and ${\tilde{\varepsilon }}_{p}$ are the complex permittivity of the suspending medium and particle respectively; and $\;\tilde{\varepsilon }=\varepsilon -\frac{j\sigma }{\omega }$, where $\;j^{2}=-1$ and ε and σ are permittivity and conductivity. The relative permittivity and conductivity of the 100 nm polystyrene beads are set as 2.55 and 7.2 mS/m, respectively [26,41,42,43,44].

The geometrical constant $G_{f}$ in Equation (4) can be presented as $G_{f}=\kappa w$ [17], where w is the width of the electrode and κ is the correction factor describing the fringing field. The value of κ was derived analytically using the conforming mapping method [17,31,45]. Utilizing this method, κ and geometric constant $G_{f}$ were calculated as 0.73 and 7.3 μm, respectively (the details of the derivation is provided in the Supplementary Information (Figure S1)).

## 3. Results and Discussion

### 3.1. Particles Entrapment

**Figure 4:**
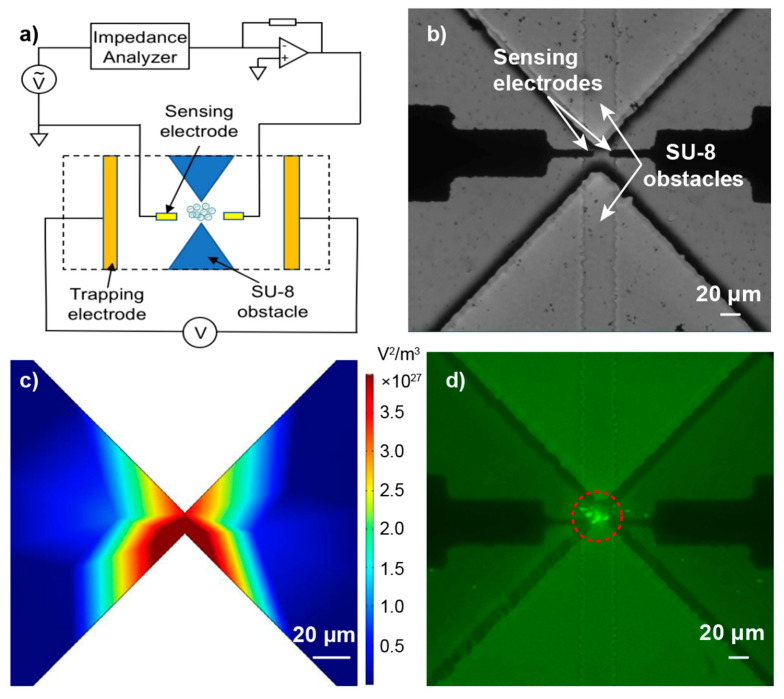
(**a**) The schematic of the impedance measurement system. (**b**) The bright-field microscopic image of the device. (**c**) The finite element analysis of the distribution of the electric field gradient across the opening created by SU-8; the suspending medium was 10 mM KCl and the applied voltage was 5 V/mm. (**d**) The fluorescence microscopic images showing the entrapment of 100 nm fluorescently tagged carboxylic acid polystyrene (COOH-PS) beads with a 5 V/mm bias applied across the opening for 5 min; the initial particle concentration was 2.3 × 10^12^/mL and the suspending solution was 10 mM KCl.

In our previous work, we have demonstrated that COOH-PS beads, liposomes, and exosomes with sub-micron diameters could be rapidly trapped at the tip of a glass micropipette due to the balance of DEP, EP, and EOF forces [26,27]. Others have also reported that the micro-pores constructed by SU-8 or PDMS triangles are effective geometrical designs to isolate particles and cells utilizing electrokinetics [46,47]. Here, to integrate the trapping mechanism with the sensing module on a single chip (Figure 4a), we developed SU-8 constructed micro-pores with 10 μm width and 50 μm height to trap particles utilizing DEP. Furthermore, a pair of co-planar electrodes (12 μm × 10 μm with 10 μm gap distance) were fabricated to measure the impedance of the trapped particles (Figure 4b). A finite element simulation was carried out to study the distribution of the electric field (E-field) gradient under DC bias (Figure 4c). The results illustrate that the highest E-field gradient was localized at the narrowest part of the opening, which was consistent with our previous study [47]. 

A series of experiments were conducted with fluorescently-tagged COOH-PS beads, fluorescently-tagged liposomes, and exosomes suspended in 10 mM KCl (pH 7.0). A 25 μL solution containing various particles were injected separately into different chambers of the device and 5 V/mm DC bias was applied across the opening for 5 min. Figure 4d and Figure S2 show that the particles were trapped at the narrowest region of the opening as expected. 

### 3.2. Impedance Measurement of Solution with Various Ionic Strengths

**Figure 5:**
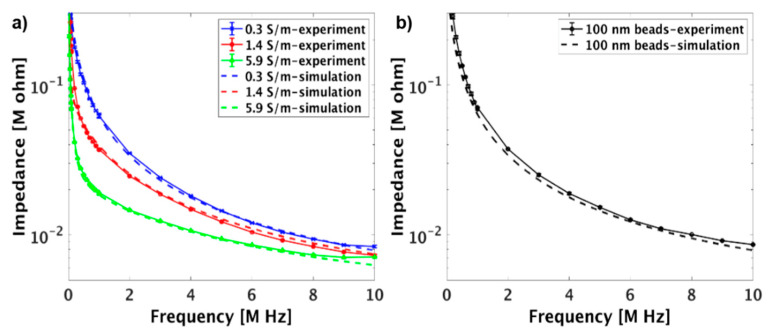
(**a**) The theoretical modeling and experimental results showing the impedance of solutions with different conductivities as a function of frequency. (**b**) The theoretical modeling and experimental results showing the impedance of COOH-PS beads suspended in 10 mM KCl. The error bars represent the standard deviation and each experiment was repeated at least three times.

To study the capability of the device to differentiate between solutions with different ionic strengths, and understand the physical principle of the impedance measurement, an equivalent circuit model was constructed and the theoretical and experimental results were compared. Figure 5a demonstrates both the theoretical and experimental results of the impedance when solutions with different conductivities were tested. The theoretical results were closely matched with the experimental measurements, which implies that the established equivalent circuit model was reliable for predicting the impedance of the system. In addition, the results were in line with the previously reported observations [48,49,50,51] and suggest that as the frequency increased, the absolute value of impedance decreased for all solutions. This is due to the fact that the reactive part of the impedance was predominately capacitive and, thus, the co-planar impedance sensor acted as a capacitor, storing electrochemical energy [49]. Statistical data obtained from the experimental measurements are shown in Table S2 indicating that the impedance of the solutions was significantly different from each other (*p* < 0.05) at a wide frequency spectrum, and thus, the device is capable of differentiating solutions with different ionic strengths. However, the results also indicate that the impedance of the solutions with 1.4 and 5.9 S/m conductivities at frequency ≥ 10 MHz were not significantly different from each other. This could be justified since the stray capacitance is dominated at frequency ≥ 10 MHz which resulted in the reduction of the difference in their impedance. [52]. In addition, to further investigate the capability of the circuit model to predict the impedance of the particles, theoretical results and experimental measurements were compared utilizing the well-defined 100 nm COOH-PS beads suspended in 10 mM KCl. Figure 5b demonstrates that the theoretical results were closely matched with the experimental measurements, which proves that the established equivalent circuit model is reliable for predicting the impedance of the system with added beads.

### 3.3. Impedance Measurements of Sub-Micron Particles in Solution

**Figure 6:**
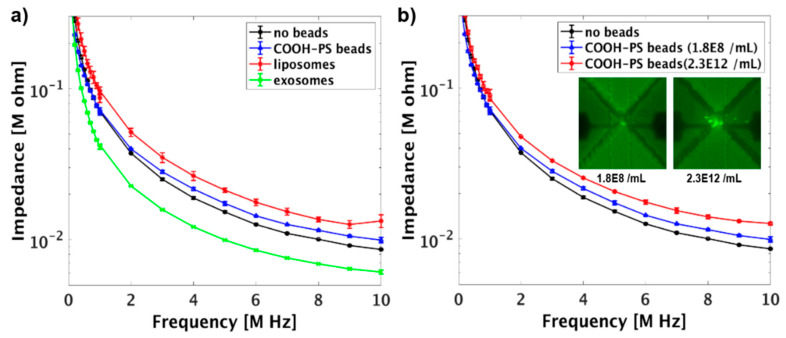
(**a**) The impedance of different particles suspended in electrolytic (10 mM KCl) solution as a function of frequency. The initial concentration of the COOH-PS beads was 1.8 × 10^8^/mL. (**b**) The impedance of the COOH-PS beads with different initial concentrations suspended in 10 mM KCl solution. The error bars represent the standard deviation and each experiment was repeated at least three times.

To investigate the impedance response of different sub-micron particles, COOH-PS beads, liposomes, and exosomes, suspended in 10 mM KCl were injected into different chambers of the device. The particles were trapped at the triangular trapping zones by applying DC bias, and their impedances were recorded under AC field. The impedance of the entrapped liposomes and COOH-PS beads were increased when compared to the solution containing no particles (Figure 6a). This result could be justified since the lipid bilayer in liposome and the bulk polystyrene materials in COOH-PS beads have lower conductivities when compared to the surrounding medium, and, thus, resulting in the enhancement of the channel resistance [53,54]. However, as exosomes were incorporated into the system, a lower impedance was measured. This result suggests that exosomes were more conductive than the suspending medium, which is because proteins with a relatively high conductivity are embedded on the exosomes’ membrane [55,56].

To further study the impedance response of the particles with different concentration, COOH-PS beads with different initial concentration (1.8 × 10^8^/mL and 2.3 × 10^12^/mL) were injected into different chambers of the LOC device and trapped by applying 5 V/mm DC field for 5 min. The microscopic images in Figure 6b show that as the initial concentration of COOH-PS beads was increased, more beads were trapped at the triangular trapping zone [26]. Consequently, the impedance of the system significantly increased due to the enhancement of the channel resistance and the reduction of channel capacitance (Figure 6b and Table S3) [53]. The impedance sensitivity calculations are shown in Table S4, which indicate that the sensitivity of the device is in the range of 0.03 to 0.55. The obtained values were similar to the sensitivity of cell impedance cytometer estimated by Sun et al. [31]. In addition, as the initial particle concentration of COOH-PS beads increased from 1.8 × 10^8^/mL to 2.3 × 10^12^/mL, the impedance sensitivity significantly increased due to the increase of the mixture impedance (the medium and the particle ) in the detection volume.

**Figure 7:**
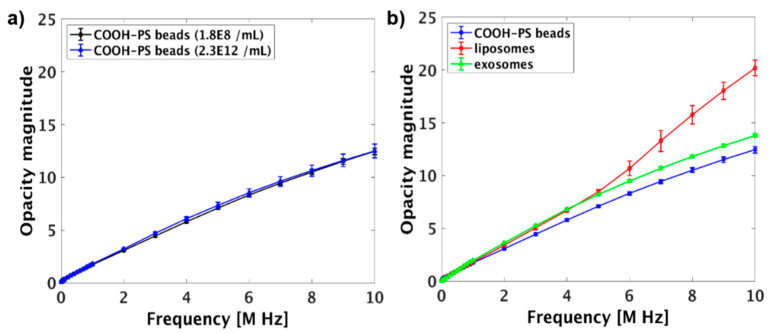
(**a**) The opacity magnitude of 100 nm COOH-PS beads with different entrapped quantities. (**b**) Opacity magnitude of different particles suspended in 10 mM KCl. The error bars represent the standard deviation and each experiment was repeated at least three times.

To rule out the effect of the particles’ concentration on their impedance and only show the effect of their dielectric properties by impedance measurements, the results were normalized based on the opacity concept [3,15,28]. The impedance of the COOH-PS beads with different initial concentration (1.8 × 10^8^/mL and 2.3 × 10^12^/mL) were normalized based on opacity magnitude and plotted in Figure 7a and summarized in Table S5. The results demonstrate that there were no significant differences (*p* > 0.05) between the opacities of COOH-PS beads with different initial concentrations. 

To further investigate the capability of the system to differentiate between particles with different dielectric properties, the opacity magnitude of three types of particles with different compositions were analyzed and plotted in Figure 7b. A detailed representation of the data with statistical analysis is shown in Table S6. The results show that COOH-PS beads and exosomes were differentiated at frequency range ≥ 1 MHz, and COOH-PS beads and liposomes were differentiated at the frequency range ≥ 2 MHz. This results indicate that the dielectric properties of the COOH-PS beads is vastly different from the nanovesicles due to the difference of composition and surface charge (Table S1) [13,29]. In addition, liposomes and exosomes could be differentiated at the frequency range ≥ 6 MHz, which most likely reflects on differences in their membrane capacitance due to the presence of proteins on the exosomes’ membrane [13,29,55].

## 4. Conclusions

In this study, we have demonstrated a microchip device which is capable of entrapment of nanovesicles utilizing an insulator-based dielectrophoretic (iDEP) module and an integrated impedance measurement system to characterize the nanovesicles based on their dielectric properties. The device is comprised of SU-8 obstacles to create micron-size openings to create a non-uniform electric field in order to entrap particles as a result of the balance of three electrokinetic forces under DC bias. The entrapped particles could be further analyzed based on their impedance by an integrated co-planar sensor under AC field and a wide frequency spectrum. The impedance of solution with different ionic strengths and the well-defined COOH-PS beads were measured to validate our empirical results and the results were matched with the theoretical equivalent circuit model. Also, the results obtained by analysis of nanoparticles (COOH-PS) and nanovesicles (liposomes and exosomes) of similar size, demonstrated that the device is capable of discriminating between different particles with different compositions and hence, different dielectric properties at a frequency range of ~2 to 10 MHz. As a result, the proposed device could potentially be applied for characterization and detection of pathogenic nanovesicles based on their unique dielectric properties, and thus, further evolved as a powerful tool for early disease diagnosis and prognosis.

## Supplementary Materials

The following are available online at https://www.mdpi.com/2072-666X/12/1/11/s1, Figure S1: Diagrams showing the conformal transformation from physical plane (x,z) to model plane (u,v). Figure S2: (a) The microscopic images of entrapped fluorescently-tagged liposomes. (b) The microscopic images of entrapped hTERT Mesenchymal Stem Cell Exosomes. A 5 V/mm bias was applied across the channel for 5 min and the suspending solution was 10 mM KCl. Table S1: Zeta potential and size of COOH-PS beads, liposomes, and exosomes. The zeta potential of particles were measured in 10 mM KCl. Table S2: The statistical data for the impedance measurement of different electrolyte solutions. *p*-values were obtained from two-tail unpaired student *t*-test. The highlighted data are significantly different. Table S3: The statistical data for the impedance measurement of different particles suspended in 10 mM KCl. *p*-values were obtained from two-tails unpaired student *t*-test. The highlighted data are significantly different. Table S4: The statistical data for the impedance sensitivity of different particles. *p*-values were obtained from two-tail unpaired student *t*-test. The highlighted data are significantly different. Table S5: The statistical data for the opacity magnitude of COOH-PS beads with different concentration suspended in 10 mM KCl. *p*-values were obtained from two-tail unpaired student *t*-test. Table S6: The statistical data for the opacity magnitude of different particles suspended in 10 mM KCl. *p*-values were obtained from two-tail unpaired student *t*-test. The highlighted data are significantly different.
