# Supplementary material for: An Electrokinetically-Driven Microchip for Rapid Entrapment and Detection of Nanovesicles
Source: Micromachines (Basel). 2020 Dec 24;12(1):11. doi: 10.3390/mi12010011 (PMC7823576; doi:10.3390/mi12010011)
Supplement: Supplementary file 1 [file micromachines-12-00011-s001.pdf]

micromachines

Supplementary Information: An Electrokinetically-Driven Microchip for Rapid Extraction and Detection of Nanovesicles

Leilei Shi [1] and Leyla Esfandiari [1,2,*]

[1] Department of Electrical Engineering and Computer Science, College of Engineering and Applied Sciences, University of Cincinnati, Cincinnati, OH 45221, USA; shili@mail.uc.edu
[2] Department of Biomedical Engineering, College of Engineering and Applied Sciences, University of Cincinnati, Cincinnati, OH 45221, USA
* Correspondence: esfandla@ucmail.uc.edu

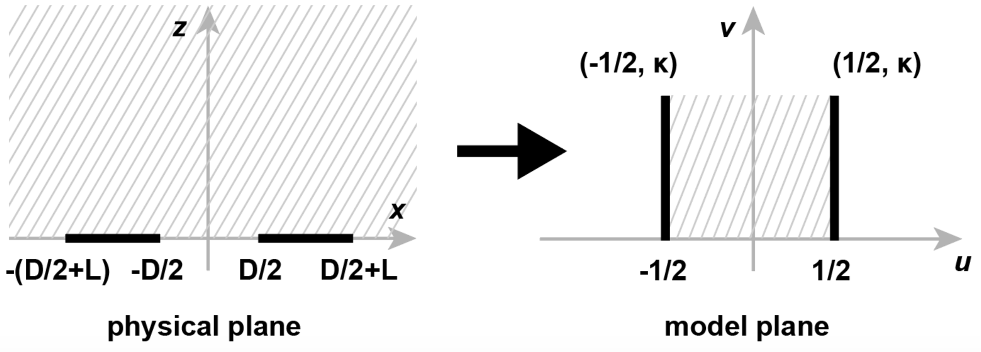

Figure S1. Diagrams showing the conformal transformation from physical plane (x,z) to model plane (u,v).

The geometric constant of the system is defined by $G_f = \kappa w$, where w is the width of the electrode and κ is the correction factor describing the fringing field. The value of κ was derived analytically using the conforming mapping method.[1-3] With this method, each particular curve or shape on the physical plane (x,z) is converted into a corresponding curve or shape on the model plane (u,v). The correction factor, κ, is given by:

$$\kappa = \frac{K(k_b')}{2K(k_b)} \tag{1}$$

where the modulus k_b is related to the electrode lay-out. Considering a pair of co-planar electrodes with a gap distance D, and electrode length L, κ could be solved based on the following equations.

$$k_b = \frac{D}{D + 2L} \tag{2}$$

$$k_b' = \sqrt{1 - k_b^2} \tag{3}$$

$$K(k_b) = \int_0^1 \frac{dt}{\sqrt{(1 - t^2)(1 - k_b^2 t^2)}} \tag{4}$$

With a gap distance of D = 10 μm, the electrode width of W = 10 μm and electrode length of L = 12 μm, cell constant per unit width κ and geometric constant G_f were calculated as 0.73 and 7.3 μm, respectively.

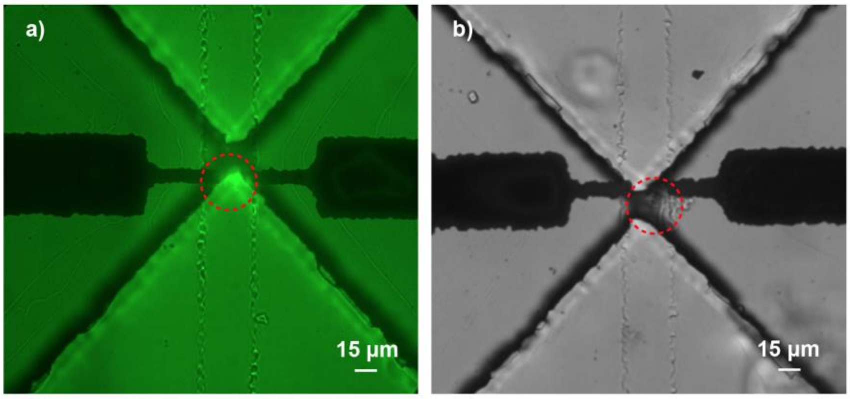

Figure S2. (**a**) The microscopic images of entrapped fluorescently-tagged liposomes. (**b**) The microscopic images of entrapped hTERT Mesenchymal Stem Cell Exosomes. A 5 V/mm bias was applied across the channel for 5 minutes and the suspending solution was 10 mM KCl.

Table S1. Zeta potential and particle size of COOH-PS beads, liposomes, and exosomes. The zeta potential was measured in 10 mM KCl.

Particles	Zeta Potential (mV)	Particle Size (nm)
COOH-PS beads	-18.25 ± 4.07	93.0 ± 24.0
Liposomes	-2.07 ± 3.85	106.6 ± 1.9
Exosomes	-7.89 ± 1.90	146.1 ± 65.4

Table S2. The statistical data for the impedance measurement of different electrolyte solutions. p-values were obtained from two-tails unpaired student t-test. The highlighted data are significantly different.

solutions / p-value / frequency	0.3 S/m vs.1.4 S/m	0.3 S/m vs. 5.9 S/m	1.4 S/m vs. 5.9 S/m
**1 MHz**	0.002110240	0.000794990	0.000001331
**2 MHz**	0.000000260	0.000000114	0.000000087
**3 MHz**	0.000004511	0.000000019	0.000000157
**4 MHz**	0.001004880	0.000029372	0.000091765
**5 MHz**	0.000199100	0.000123430	0.000015154
**6 MHz**	0.000846810	0.000020085	0.000040799
**7 MHz**	0.000014075	0.000002286	0.000044813
**8 MHz**	0.000009807	0.000002244	0.000017055
**9 MHz**	0.000695230	0.000319000	0.000116890
**10 MHz**	0.000498190	0.003033120	0.112107950

Table S3. The statistical data for the impedance measurement of different particles suspended in 10 mM KCl. p-values were obtained from two-tails unpaired student t-test. The highlighted data are significantly different.

particles / p-value / frequency	COOH-PS (1.8E8/mL) vs. no beads	COOH-PS (2.3E12/mL) vs. no beads	liposome vs. no beads	exosomes vs. no beads
1 MHz	0.898068747	0.042732242	0.034060472	0.000016928
2 MHz	0.000694623	0.000003608	0.016308972	0.000000867
3 MHz	0.003643737	0.000023126	0.019744964	0.000258871
4 MHz	0.003235402	0.000011857	0.015806760	0.000265573
5 MHz	0.012290758	0.000011476	0.002682736	0.000427755
6 MHz	0.000101575	0.003625013	0.009799502	0.000004183
7 MHz	0.000057251	0.005400913	0.011102776	0.000002319
8 MHz	0.004704614	0.003443929	0.006438732	0.000000165
9 MHz	0.005256587	0.000587598	0.014438355	0.000215623
10 MHz	0.030149983	0.001058547	0.023403918	0.000585922

Table S4. The statistical data for the impedance sensitivity of different particles. p-values were obtained from two-tails unpaired student t-test. The highlighted data are significantly different.

Frequency (MHz)	Particles	Impedance Sensitivity		P-value			
		Mean	SD	COOH-PS (1.8E8 /mL)	COOH-PS (2.3E12 /mL)	liposome	exosome
1	COOH-PS (1.8E8 /mL)	0.033132529	0.03359412	1			
	COOH-PS (2.3E12 /mL)	0.221706987	0.04068923	0.003886223	1		
	liposome	0.379378677	0.08143311	0.009389681	0.059167556	1	
	exosome	0.397361586	0.00710482	0.00197903	0.015208365	0.7393538	1
2	COOH-PS (1.8E8 /mL)	0.071096878	0.00556061	1			
	COOH-PS (2.3E12 /mL)	0.275433191	0.00423581	1.93671E-06	1		
	liposome	0.377502728	0.08474698	0.024154405	0.171967586	1	
	exosome	0.393621685	0.0006981	7.85071E-05	0.000310683	0.7731302	1
3	COOH-PS (1.8E8 /mL)	0.123095281	0.02488869	1			
	COOH-PS (2.3E12 /mL)	0.319212901	0.01612993	0.000748966	1		
	liposome	0.399547411	0.10363063	0.037569143	0.31062628	1	
	exosome	0.370018957	0.00559854	0.002297258	0.022362253	0.6706876	1
4	COOH-PS (1.8E8 /mL)	0.129913173	0.02720364	1			
	COOH-PS (2.3E12 /mL)	0.327240824	0.01534086	0.001296144	1		
	liposome	0.385473522	0.09391465	0.033846039	0.395432362	1	
	exosome	0.364805286	0.00479482	0.003560736	0.041250055	0.7399	1
5	COOH-PS (1.8E8 /mL)	0.11959612	0.0362081	1			
	COOH-PS (2.3E12 /mL)	0.32967964	0.01587285	0.003862563	1		
	liposome	0.37319396	0.04893439	0.002662989	0.259682306	1	
	exosome	0.35896001	0.00336801	0.007149515	0.079673231	0.664699	1
6	COOH-PS (1.8E8 /mL)	0.127187458	0.00926207	1			
	COOH-PS (2.3E12 /mL)	0.375274306	0.04801739	0.010139392	1		
	liposome	0.385404053	0.07250144	0.023763636	0.851362193	1	
	exosome	0.331325967	0.00867351	1.03E-05	0.251647413	0.3251007	1
7	COOH-PS (1.8E8 /mL)	0.135822528	0.00482014	1			
	COOH-PS (2.3E12 /mL)	0.389637568	0.05462033	0.014532434	1		
	liposome	0.379955549	0.0738384	0.028722787	0.864722015	1	
	exosome	0.31879175	0.00554673	2.12E-06	0.152386483	0.2875057	1
8	COOH-PS (1.8E8 /mL)	0.141339892	0.02004531	1			
	COOH-PS (2.3E12 /mL)	0.392189398	0.04200296	0.003125319	1		
	liposome	0.350498562	0.05070408	0.010576338	0.336325806	1	
	exosome	0.315611478	0.00310855	0.00368734	0.086548933	0.2143704	1
9	COOH-PS (1.8E8 /mL)	0.141339892	0.02074872	1			
	COOH-PS (2.3E12 /mL)	0.392189398	0.021088	7.26645E-05	1		
	liposome	0.350498562	0.08074649	0.031823571	0.345306155	1	
	exosome	0.315611478	0.01182467	0.001343835	0.001712376	0.2143704	1
10	COOH-PS (1.8E8 /mL)	0.154498833	0.04860931	1			
	COOH-PS (2.3E12 /mL)	0.470923361	0.03253359	0.001349617	1		
	liposome	0.548283849	0.14799633	0.033718256	0.46268372	1	
	exosome	0.291033889	0.0203904	0.026091074	0.002545376	0.0922136	1

Table S5. The statistical data for the opacity magnitude of COOH-PS beads with different concentration suspended in 10 mM KCl. p-values were obtained from two-tails unpaired student t-test.

frequency \ p-value \ particles	COOH-PS (1.8E8/mL) vs. COOH-PS (2.3E12/mL)
**1 MHz**	0.465098099
**2 MHz**	0.078113122
**3 MHz**	0.076516641
**4 MHz**	0.148602211
**5 MHz**	0.289409709
**6 MHz**	0.429568421
**7 MHz**	0.575761658
**8 MHz**	0.708662559
**9 MHz**	0.809743316
**10 MHz**	0.896077618

Table S6. The statistical data for the opacity magnitude of different particles suspended in 10 mM KCl. p-values were obtained from two-tails unpaired student t-test. The highlighted data are significantly different.

frequency \ p-value \ particles	liposome vs. exosomes	COOH-PS vs. liposome	COOH-PS vs. exosomes
**1 MHz**	0.076102190	0.088997750	0.015013160
**2 MHz**	0.089565580	0.004401860	0.003014760
**3 MHz**	0.180260390	0.020341510	0.000627480
**4 MHz**	0.424774700	0.004770360	0.000490200
**5 MHz**	0.234101400	0.002481550	0.000556540
**6 MHz**	0.096276430	0.023067910	0.000798800
**7 MHz**	0.043149860	0.018634060	0.001281590
**8 MHz**	0.012611930	0.005928300	0.002106360
**9 MHz**	0.005350830	0.002331010	0.003387370
**10 MHz**	0.002473570	0.000882300	0.005091060

References

1. Hong, J.; Yoon, D.S.; Kim, S.K.; Kim, T.S.; Kim, S.; Pak, E.Y.; No, K. AC frequency characteristics of coplanar impedance sensors as design parameters. *Lab Chip* **2005**, *5*, 270–279.
2. Sun, T.; Green, N.G.; Gawad, S.; Morgan, H. Analytical electric field and sensitivity analysis for two microfluidic impedance cytometer designs. *IET nanobiotechnology* **2007**, *1*, 69–79.
3. Sun, T.; Bernabini, C.; Morgan, H. Single-colloidal particle impedance spectroscopy: Complete equivalent circuit analysis of polyelectrolyte microcapsules. *Langmuir* **2009**, *26*, 3821–3828.
